# 373. Household transmission of SARS-CoV-2 B.1.1.7 lineage –2 U.S. States, 2021

**DOI:** 10.1093/ofid/ofab466.574

**Published:** 2021-12-04

**Authors:** Raymond Soto, Christoper Hsu, Meagan Chuey, Marisa Donnelly, Victoria T Chu, Noah G Schwartz, Suxiang Tong, Natalie J Thornburg, Marie E Killerby, J Erin Staples, Hannah L Kirking, Jacqueline Tate, Almea Matanock, Ginger Stringer, Bernadette Albanese, Mark Beatty, Laura J Hughes

**Affiliations:** 1 Arboviral Diseases Branch, Centers for Disease Control and Prevention, Fort Collins, Colorado; 2 CDC, Atlanta, Georgia; 3 Centers for Disease Control and Prevention, San Deigo, California; 4 Division of Viral Diseases, Centers for Disease Control and Prevention (CDC), Atlanta, Georgia; 5 U.S. CDC, Ft. Collins, Colorado; 6 US Centers for Disease Control and Prevention, Atlanta, Georgia; 7 Division of Viral Diseases, Centers for Disease Control and Prevention, Atlanta, GA; 8 Colorado Department of Public Health and Environment, Denver, Colorado; 9 Tri-County Health Department, Greenwood Village, Colorado; 10 County of San Diego Health and Human Services Agency, San Diego, California

## Abstract

**Background:**

In December 2020, B.1.1.7 lineage of SARS-CoV-2 was first detected in the United States and has since become the dominant lineage. Previous investigations involving B.1.1.7 suggested higher rates of transmission relative to non-B.1.1.7 lineages. We conducted a household transmission investigation to determine the secondary infection rates (SIR) of B.1.1.7 and non-B.1.1.7 SARS-CoV-2 lineages.

**Methods:**

From January–April 2021, we enrolled members of households in San Diego County, CA, and Denver, CO metropolitan area (Tri-County), with a confirmed SARS-CoV-2 infection in a household member with illness onset date in the previous 10 days. CDC investigators visited households at enrollment and 14 days later at closeout to obtain demographic and clinical data and nasopharyngeal (NP) samples on all consenting household members. Interim visits, with collection of NP swabs, occurred if a participant became symptomatic during follow-up. NP samples were tested for SARS-CoV-2 using TaqPath™ RT-PCR test, where failure to amplify the spike protein results in S-Gene target failure (SGTF) may indicate B.1.1.7 lineage. Demographic characteristics and SIR were compared among SGTF and non-SGTF households using two-sided p-values with chi-square tests; 95% confidence intervals (CI) were calculated with Wilson score intervals.

**Results:**

552 persons from 151 households were enrolled. 91 (60%) households were classified as SGTF, 57 (38%) non-SGTF, and 3 (2%) indeterminant. SGTF and non-SGTF households had similar sex distribution (49% female and 52% female, respectively; P=0.54) and age (median 30 years, interquartile range (IQR 14–47) and 31 years (IQR 15–45), respectively). Hispanic people accounted for 24% and 32% of enrolled members of SGTF and non-SGTF households, respectively (p=0.04). At least one secondary case occurred in 61% of SGTF and 58% of non-SGTF households (P=0.66). SIR was 52% (95%[CI] 46%-59%) for SGTF and 45% (95% CI 37%-53%) for non-SGTF households (P=0.18).

**Conclusion:**

SIRs were high in both SGTF and non-SGTF households; our findings did not support an increase in SIR for SGTF relative to non-SGTF households in this setting. Sequence confirmed SARS-CoV-2 samples will provide further information on lineage specific SIRs.

**Disclosures:**

**All Authors**: No reported disclosures

